# Factors Associated with Primary Care, Behavioral Health, and Emergency Department Utilization Among Men with Opioid Use Disorder and Criminal-Legal Involvement: A Cross-Sectional Study

**DOI:** 10.21203/rs.3.rs-9536483/v1

**Published:** 2026-05-08

**Authors:** Danta D. R. Bien-Aimé, Sarah Mazen, Cassange Bitere, Gareth Parry, Ana M. Progovac, Michael William Flores

**Affiliations:** Cambridge Health Alliance; Cambridge Health Alliance; Emory University; Cambridge Health Alliance; New York University; Cambridge Health Alliance

**Keywords:** Opioid Use Disorder, Healthcare Utilization, Criminal-legal Involvement, Behavioral Health, Safety-Net

## Abstract

**Background:**

Men with both criminal-legal involvement (CLI) and opioid use disorder (OUD) experience high overdose risk and substantial barriers to evidence-based treatment. However, the intersection of both conditions remains understudied, and less is known about factors associated with healthcare utilization among individuals with OUD and CLI. This study examined individual- and neighborhood-level factors associated with service utilization among this population.

**Methods:**

We conducted a cross-sectional analysis of a de-identified, linked dataset (2015–2019) combining electronic health records from an urban safety-net healthcare system, local prison records, and neighborhood-level data. The sample included adult men with both CLI and documented OUD (n = 159). Outcomes were any primary care, behavioral health, and emergency department (ED) use. Guided by the Andersen Behavioral Model for Vulnerable Populations, we estimated Firth bias-reduced logistic regression models.

**Results:**

Over the study period, 64.8% had at least one primary care visit, 62.9% had at least one behavioral health visit, and 95.0% had at least one ED visit. Need-related factors showed the most consistent associations with service use. Anxiety disorders and non-opioid substance use disorders were associated with higher odds of both primary care and behavioral health use; Few factors were associated with ED utilization. Predisposing and enabling factors, including age, race/ethnicity, and insurance type, were not significantly associated with service use.

**Conclusions:**

In this safety-net population, healthcare utilization was common and was primarily associated with behavioral health need rather than predisposing or enabling factors. ED use was very common and not strongly associated with measured individual or neighborhood-level factors, suggesting that emergency care may function as a point of entry into the healthcare system for this population. These findings suggest that, in this high-risk population, clinical need may play a more important role in influencing service use than traditional structural factors.

## 1. INTRODUCTION

The opioid epidemic has long been a major public health emergency in the United States for over twenty-five years(1) and was officially declared a national public health crisis in 2017, with renewed federal calls to action in 2024 (2). In 2022 alone, opioids accounted for approximately 76% of all fatal drug overdoses (3), with synthetic opioids, mainly illegally manufactured fentanyl and its analogues, responsible for 90% of them (1). According to the World Health Organization (WHO), men and people of low socio-economic status are most at risk of opioid overdose,(4) two groups that experience unique yet overlapping challenges, including housing instability and limited resources. Additionally, people with a history of criminal-legal involvement (CLI) face additional risks(5–7), with drug overdose a leading cause of death among this group (7).

During incarceration, individuals have limited access to treatment and social supports (8). Upon release, the risk of overdose death is approximately ten times higher than in the general population (9). Formerly incarcerated individuals often face multiple and competing challenges–such as securing health insurance coverage, employment, housing, and access to mental health and substance use services–which can further prevent their recovery (10–12). These barriers, combined with the period of forced abstinence during incarceration, increase relapse risk and contribute to cyclical healthcare utilization, often marked by fragmented contact with emergency departments (EDs) rather than sustained engagement in primary or behavioral care (13, 14). Men, who represent the vast majority (93.3%) of the incarcerated population, are especially vulnerable to these intersecting risks (15).

A crucial way to reduce overdose risk and facilitate engagement with Substance Use Disorder (SUD) treatment is for people with CLI and Opioid Use Disorder (OUD) to have access to primary care (16). As a critical intervention point, primary care improves OUD diagnosis (17) and increases access to medication-assisted treatment for opioid use disorder (MOUD) (18–20), which can reduce the risk of fatal overdose by up to 62% (21). It also connects patients to behavioral health and recovery support services, and helps address the social determinants of health that contribute to OUD (22, 23). Behavioral health services, in turn, are often an alternative or parallel entry point for individuals with complex needs who experience stigma, criminalization, or fragmented access to medical care (24, 25). Conversely, reliance on emergency departments has been described as reflecting gaps in preventive and outpatient service systems and delayed or crisis-driven care (26).

Existing research shows that people with substance use disorder (OUD) or those with criminal-legal system involvement experience substantial barriers to service utilization, influenced by predisposing, enabling, and need-related factors. Studies applying the Andersen and Gelberg–Andersen Behavioral Model to vulnerable populations (27) show that predisposing factors, such as younger age, male sex, and racial/ethnic minority status, are associated with lower primary care use and increased reliance on emergency departments (EDs) (8, 28, 29). Enabling factors, including unstable housing, lack of transportation, limited income, and health insurance coverage after release from incarceration, decrease access to outpatient services and continuity of care (30). Need-related factors such as co-occurring mental health disorders, chronic physical conditions, and prior overdose events increase both behavioral health and emergency department use (31).

Although prior research has investigated service use among CLI individuals or among those with OUD (32–34), few studies have specifically focused on their intersection, particularly in safety-net settings, which are critical care access points for underserved and minoritized populations (35, 36). Understanding the factors that influence primary, behavioral, and emergency care utilization among this high-risk population is essential to guide interventions that improve continuity of care, reduce avoidable ED use, and prevent morbidity and premature mortality.

Guided by the Andersen Behavioral Model for Vulnerable Populations (37), this study examines predisposing, enabling, and need-related factors associated with primary care, behavioral health, and emergency department service use among men with CLI and a documented OUD diagnosis.

The conceptual framework, adapted from the Gelberg-Andersen Behavioral Model (38, 39), accounts for the complex and unique needs of individuals who are at increased risk for poor health outcomes due to social, economic, or behavioral determinants (37, 40). The model categorizes the determinants of service utilization into three domains: (1) *Predisposing factors*—demographic and social characteristics that influence the likelihood of service use; (2) *Enabling factors*—resources that facilitate or hinder access (e.g., income, health insurance); and (3) *Need factors*—perceived or evaluated health conditions that motivate service use (41, 42). It also distinguishes between individual-level and community-level (contextual factors) influences (43, 44). This framework is widely used to study service utilization among populations experiencing homelessness, mental illness, and substance use disorders (27, 41, 42), and is increasingly applied to incarcerated or CLI populations (28, 45).

## 2. METHODS

### 2.1. Design, Setting, and Sampling

This study is a secondary analysis of a de-identified, linked dataset (46) constructed from electronic health records (EHR) of an urban safety-net healthcare system in the Northeast, local prison administrative records, and neighborhood-level data from the 2015–2019 American Community Survey (47). The dataset includes individual-level demographics and clinical variables, block group–level characteristics, healthcare service utilization, behavioral health diagnoses, and arrest history (including dates and offense types).

The study population included male adults (≥ 18 years) with a documented history of CLI and a diagnosis of OUD who had some interaction with the healthcare system during the study window (n = 159). We focused on any documented primary, behavioral, or emergency care utilization between 2015 and 2019, regardless of time since release, to capture a broader picture of service use that may occur at any time, especially given how difficult it is for this population to access services.

This study used de-identified data obtained through data use agreements with participating institutions and did not involve direct interaction with participants. The study did not constitute human subjects research; therefore, institutional review board approval and informed consent were not required. Data are not publicly available due to privacy and HIPAA restrictions.

### 2.2. Main Outcomes

The key outcomes of interest were dichotomous service utilization outcomes, consisting of any primary care (yes/no), any behavioral health (yes/no), including inpatient or outpatient services, and any emergency department (ED, yes/no) between 2015 and 2019.

### 2.3. Independent measures

**Figure 1:**
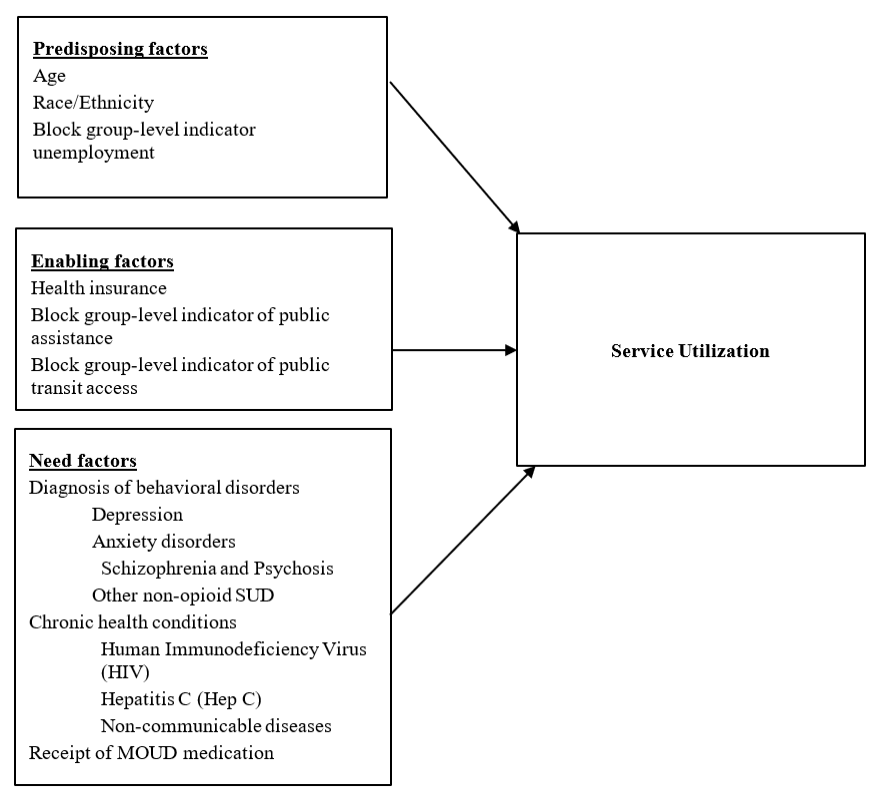
Conceptual framework

Guided by the Andersen Behavioral Model for Vulnerable Populations (37), we examined a set of individual- and neighborhood-level characteristics that prior work has identified as among the most salient factors associated with service utilization among people with substance use disorder (48–50). At the individual level, we prioritized demographics, insurance coverage, behavioral and chronic health comorbidities, reflecting factors that both influence access to care and directly capture clinical needs. At the neighborhood (block group) level, we selected indicators of local economic disadvantage and transit access, which are consistently linked to service use (51). We selected block group–level variables to capture more granular and localized indicators that may better reflect the environments in which individuals access care. Our primary objective was to examine how these theoretically and empirically salient determinants are associated with primary care, behavioral health, and emergency department use among men with OUD and CLI (Fig. 1).

#### 2.3.1. Predisposing factors

Predisposing factors included age and race/ethnicity, both commonly associated with patterns of healthcare utilization in the general population and among people with substance use disorders or criminal-legal involvement (28, 52). At the neighborhood level (block group), we included an indicator of unemployment (percentage of unemployed individuals aged 16 and older). Unemployed individuals were reported to be at a greater risk of being uninsured and unable to afford care (53, 54). We used this variable as an indicator of local economic disadvantage that may be directly linked to barriers to care.

#### 2.3.2. Enabling factors

Individual-level enabling factors included health insurance type (private vs. public insurance, where public insurance included Medicare or Medicaid plans). Individuals without insurance or those coded as self-pay were excluded from our analyses due to small sample size. Health insurance is a well-established enabling determinant of service use. Prior studies show that people with Medicaid have higher ED use and more fragmented primary care due to unmet needs (55). Medicaid significantly increases behavioral health service use and Medications for Opioid Use Disorders (MOUD) initiation (56).

Neighborhood-level enabling factors included at the block group-level: (1) an indicator of public assistance (the percentage of households receiving public assistance income, food stamps or SNAP in the past 12 months) and (2) an indicator of public transit access (the percentage of workers commuting by public transit). Household receipt of public assistance reflects engagement with social safety-net programs and may enable healthcare access through greater linkage to public health systems and social service supports, which in turn may influence service utilization (57). Transit access reflects the availability of transportation infrastructure, an essential structural facilitator of healthcare use, particularly in low-income urban communities (58). It is a well-documented determinant of service utilization, particularly for individuals with SUD and other chronic conditions; greater transit access is associated with higher outpatient and behavioral health use and better MOUD retention (59).

#### 2.3.3. Need-related factors

Need-related factors were measured at the individual level and included binary indicators of behavioral health diagnoses (depression, anxiety, schizophrenia/psychosis, and other substance use disorders), chronic health conditions (hepatitis C, HIV, and noncommunicable conditions), and receipt of medications for MOUD (methadone, buprenorphine, naltrexone). These conditions are associated with increased health service use, including primary care, specialty care, and emergency services (60, 61). Noncommunicable chronic diseases were included because people with OUD and CLI often have unmet preventive and chronic care needs due to fragmented healthcare use, structural disadvantage, and cumulative stress exposure (62, 63). HCV and HIV were selected because individuals with OUD are at a significantly increased risk of contracting these infectious diseases due to shared injection equipment and other risky behaviors associated with substance use (64). Receipt of MOUD was included as a need-related factor because MOUD initiation reflects clinically recognized OUD severity and provides descriptive insight into the extent to which individuals received evidence-based treatment within the healthcare system (21, 65). It was not included in the regression model because MOUD can only be prescribed after a patient has already accessed care and therefore reflects treatment received during a visit rather than a factor that influences whether a visit occurs.

### 2.4. Statistical Analysis

Among men with CLI, we first compared characteristics by OUD status using chi-square tests for categorical variables and t-tests for continuous variables.

To estimate associations between individual/neighborhood-level factors and our three binary outcomes, we used Firth bias-reduced logistic regression (penalized maximum likelihood estimation) for small samples. This method reduces small-sample bias and produces stable estimates and confidence intervals when covariate categories have few observations or complete separation (66, 67). This approach is appropriate for studies focusing on marginalized populations with low event counts (67, 68). Missingness across variables ranged from 6% to 18%. Given the relatively small sample size, sparse data, and several low-prevalence binary covariates, we prioritized model stability and used complete case analysis in combination with Firth bias-reduced logistic regression. Not all variables included in the descriptive analyses were included in the regression model; final variables were selected based on theoretical relevance, variability in the data, and model stability considerations. HIV, hepatitis C, and non-communicable chronic diseases were collapsed into a single binary indicator of chronic health conditions, consistent with the need domain of the Andersen Behavioral Model.

All tests were two-sided, and statistical significance was defined as p < 0.05. We performed all analyses using Stata/MP version 19.5 (69).

## 3. RESULTS

Table 1 presents the characteristics of men with criminal-legal involvement by OUD status. Men with both CLI and OUD were significantly more likely to be White (86% vs 50%) and to have public insurance (91% vs 77%) compared with men with CLI without OUD. They also had higher prevalence of behavioral health disorder diagnoses, such as depression (53% vs 22%), as well as higher rates of hepatitis C (12% vs 2%). Age and most neighborhood-level characteristics did not differ significantly between groups. Among men with CLI and OUD, receipt of medications for opioid use disorder (MOUD) was rare, with only 2.52% of the population receiving MOUD during the study period.

Table 2 presents service utilization among men with criminal-legal involvement by OUD status. Over the 5-year study period, nearly two-thirds of men with documented OUD and CLI (64.8%) had at least one primary care encounter. Men with OUD were significantly more likely than those without OUD to have used primary care (64.8% vs. 42.7%), behavioral health services (62.9% vs. 31.1%), and emergency department care (95.0% vs. 81.7%). Emergency department utilization was particularly common among men with OUD, with nearly all having at least one ED visit during the study period.

Table 3 presents adjusted associations between individual- and block group–level factors and service utilization among men with both criminal-legal involvement and a documented OUD diagnosis. For primary care use, diagnoses of anxiety disorders (OR = 8.19, 95% CI 2.71–24.77) and non-opioid substance use disorders (OR = 4.60, 95% CI 1.59–13.33) were associated with substantially higher odds of utilization, corresponding to approximately 719% and 360% higher odds, respectively. Living in neighborhoods with a greater percentage of residents receiving public assistance was also associated with higher odds of primary care use (OR = 1.02, 95% CI 1.00–1.03). For behavioral health service use, anxiety disorders (OR = 23.98, 95% CI 6.29–91.44) and other non-opioid substance use disorders (OR = 6.19, 95% CI 2.02–18.92) were associated with higher odds of utilization. In contrast, few factors were associated with emergency department use. The presence of chronic health conditions was associated with lower ED utilization (OR = 0.08, 95% CI 0.008–0.77); none of the other covariates showed associations with emergency department use. Predisposing and enabling factors, including age, race/ethnicity, and insurance type, were not significantly associated with any service type.

## 4. DISCUSSION

In this sample of men with both opioid use disorder (OUD) and criminal-legal involvement (CLI), a substantial proportion had used primary care or behavioral health services, and nearly all had at least one encounter with the emergency department, suggesting that this population does have some level of contact with the healthcare system. However, the low rate of MOUD suggests a gap between service contact and delivery of evidence-based treatment. Limited MOUD uptake may reflect persistent barriers such as stigma, restrictive prescribing capacity, and the under-capture of methadone treatment in EHR data (70, 71). The high rate of ED utilization may reflect fragmented or crisis-driven patterns of healthcare use (72, 73). Moreover, emergency department use was not strongly associated with individual or neighborhood-level factors, which suggests that emergency care may function as a default point of entry into the healthcare system, rather than being influenced by individual characteristics.

When examining factors associated with service utilization, need-related conditions, specifically a diagnosis of anxiety disorders and co-occurring non-opioid substance use disorders, showed the most consistent associations with both primary care and behavioral health use, whereas predisposing and most enabling factors were not significantly associated with service utilization. This pattern suggests that, within this population, healthcare utilization may be more strongly influenced by clinical needs than by traditional predisposing or enabling factors such as insurance status, race, or neighborhood characteristics. The strong association between co-occurring behavioral health conditions and service utilization is consistent with prior studies showing that individuals with behavioral and substance use disorders have more frequent interactions with the healthcare system (74). This may be because these individuals have more complex health needs and therefore require more frequent care. It is also possible that they are referred to primary care from within the same system, which increases their chances of being connected to primary care due to their behavioral health condition. These findings are consistent with the concept of a treatment-seeking effect, in which mental health symptoms motivate individuals to access care or facilitate referral pathways across service systems (75).

Among contextual factors, residence in neighborhoods with higher levels of public assistance was associated with greater primary care use. This finding may indicate that individuals living in communities more connected to social safety-net programs are more likely to be linked to healthcare services, either through co-located resources, care coordination, or community-based referral networks. In contrast, most predisposing and enabling factors, including age, race/ethnicity, insurance type, transit access, and neighborhood-level measures of public transit access and unemployment, were not associated with service use. This may reflect the relatively homogeneous socioeconomic and insurance profile of this safety-net population, in which the majority of individuals were publicly insured and already connected to care. In this context, traditional access barriers may have less explanatory power for differences in service use because structural disadvantage is common across the sample. Instead, variation in utilization appeared to be influenced primarily by differences in clinical needs. Moreover, safety-net systems often provide co-located services and established referral pathways, which may reduce the influence of demographic and insurance-related factors that are more strongly associated with access in the general population. These findings suggest that once individuals with OUD and CLI interact with the healthcare system, their service utilization patterns may be more influenced by behavioral health needs than by predisposing or enabling characteristics.

This study contributes to the literature in several ways. First, it addresses a critical gap by focusing on individuals with both OUD and criminal-legal involvement, two factors independently linked to health disparities and poor access but rarely examined together in relation to service use. Second, by applying the Andersen Behavioral Model and distinguishing between individual and contextual factors, it provides a clearer understanding of which domains most strongly influence healthcare utilization. Third, by examining primary care, behavioral health, and emergency department use, this study highlights service utilization patterns across services and demonstrates that high levels of service utilization co-occurred with low documented receipt of MOUD in this sample. In doing so, it brings attention from lack of healthcare contact alone to potential missed opportunities for treatment within systems where patients are already engaged. Finally, the use of linked electronic health records, criminal-legal, and neighborhood-level data offers a multilevel perspective on service use that is rarely available for this population. Overall, our findings suggest important areas for future research on how healthcare approaches could be integrated, especially for individuals who face intersecting challenges related to mental illness, substance use, and criminal-legal involvement.

## 5. LIMITATIONS

Our study has several limitations. First, while all individuals in our sample had a documented incarceration history, we could not establish the precise timing of healthcare service use relative to incarceration or reentry. The lack of information about the timing of incarceration, diagnosis, and service use makes it difficult to assess what happens during reentry or the sequence of care engagement. However, the study provides some cross-sectional evidence on overall patterns of service utilization among men with both OUD and criminal-legal involvement, a population that is often difficult to study using EHR linked data.

Second, the study was restricted to male individuals receiving care within a single urban healthcare system, which may limit its generalizability. Females were excluded from the analytic sample due to the incarceration facility solely housing male individuals. In addition, women represent a much smaller proportion of the incarcerated population (approximately 6–7%)(76) and have distinct pathways to opioid use and treatment engagement that would require separate contextualization (77, 78). Limiting the analysis to men provides a more homogeneous analytic cohort.

Third, the analytic sample was relatively small, resulting in wide confidence intervals and limited statistical power to detect smaller associations. To address this limitation, we used Firth bias-reduced logistic regression, which is specifically designed for small samples and sparse data and allowed us to obtain stable and interpretable estimates.

Fourth, while we incorporated neighborhood-level data, these may not fully capture the lived experiences of access, such as stigma, provider discrimination, or perceived quality of care (79). Our analysis was constrained by the limited availability of localized data. Several important factors, such as provider availability, perceived health status, and chronic health conditions, were only available at larger geographic levels. There are many prior studies that rely on county-level measures (54, 80), but the use of block group-level data in this study provides a more granular approach. This highlights a critical need for public health and policy datasets to be disaggregated and made publicly available at more localized levels (e.g., block group or census tract), especially to support research on vulnerable and underserved populations.

Finally, our findings should be interpreted within the context of the study population. The individuals included in this analysis represent a subset of men with criminal-legal involvement who were diagnosed with OUD and, therefore, had at least some interaction with the healthcare system. This group likely differs from the broader population of CLI men with undiagnosed OUD, who remain disconnected from care and may face even greater structural barriers to diagnosis and treatment. Thus, these findings likely reflect factors associated with service utilization among individuals already linked to care, rather than determinants of initial access to care. Future research should examine pathways to diagnosis and early identification among individuals with both OUD and criminal-legal involvement who remain outside the healthcare system.

## 6. CONCLUSION

In this study of service utilization among men with opioid use disorder and criminal-legal involvement within an urban safety-net system, clinical needs were primarily associated with service use rather than demographic or structural factors. We found co-occurring behavioral health disorders, particularly anxiety and other substance use disorders, as consistent factors associated with primary care and behavioral health service use. In contrast, enabling and neighborhood-level contextual variables showed limited influence once individual needs were accounted for. These results highlight how behavioral health needs contribute to shaping service utilization and suggest areas for future research on care delivery in this population. Efforts to expand access to medications for opioid use disorder remain critical. Further research is needed to better capture structural barriers to care by using more individual-level data specific to vulnerable populations. There is a need for public health data to be made available at more granular geographic levels. Future research should also examine longitudinal pathways of care engagement and explore interventions that bridge behavioral health, primary care, and social services to reduce preventable morbidity and mortality in this high-risk population.

## Figures and Tables

**Table 1:** Descriptive Characteristics Among Individuals with Criminal Legal Involvement by Opioid Use Disorder (OUD) Status (N = 531)

Sample population	OUD (n = 159)	No OUD (n = 372)	Total (n = 531)	p-value
**Predisposing factors**				
Age, n (Mean ± SD)	159 (38.47 ± 10.13)	372 (37.09 ± 13.04)	531 (37.51 ± 12.25)	0.19
Race, n (%)				
White	137 (86.16%)	186 (50.00%)	323 (60.83%)	< 0.001
Non-White	22 (13.84%)	186 (50.00%)	208 (39.17%)	
Block group-level indicator unemployment, n (Mean ± SD)	150 (7.23 ± 12.43)	305 (7.23 ± 9.55)	455 (7.23 ± 10.57)	0.99
**Enabling/Disabling factors**				
Health Insurance Type, n (%)				
Private	14 (8.86%)	79 (22.83%)	93 (18.45%)	< 0.001
Public	144 (91.14%)	267 (77.17%)	411 (81.55%)	
Block group-level indicator of public assistance, n (Mean ± SD)	154 (50.51 ± 35.65)	343 (57.82 ± 42.13)	497 (55.55 ± 40.34)	0.04
Block group-level indicator of workers commuting by public transit, n (Mean ± SD)	148 (24.71 ± 16.31)	304 (26.84 ± 15.81)	452 (26.14± 15.98)	0.18
**Need factors**				
Behavioral Health Disorders, n (%)				
Depression	85 (53.46%)	81 (21.77%)	166 (31.26%)	< 0.001
Anxiety disorders	68 (42.77%)	43 (11.56%)	111 (20.09%)	< 0.001
Psychosis and Schizophrenia	14 (8.81%)	25 (6.72%)	39 (7.34%)	0.39
Non-OUD substance use disorder	76 (47.8%)	109 (29.3%)	185 (34.84%)	< 0.001
Chronic conditions, n (%)				
HIV	3 (1.89%)	4 (1.08%)	7 (1.32%)	0.45
Hepatitis C	19 (11.95%)	6 (1.61%)	25 (4.71%)	< 0.001
Non-communicable diseases	17 (10.69%)	24 (6.45%)	41 (7.72%)	0.09
Receipt of MOUD (medications for opioid use disorder), n (%)	4 (2.52%)	3 (0.81%)	7 (1.32%)	0.11

OUD = Opioid Use Disorder; SD = Standard Deviation; HIV = Human Immunodeficiency Virus; Hep C = Hepatitis C

**Table 2:** Service utilization Among Individuals with Criminal Legal Involvement by Opioid Use Disorder (OUD) Status (N = 531)

Service use	OUD (N = 159)	No OUD (N = 372)	Total (n = 531)	p-value
**Primary Care** (%)				
Yes	103 (64.78%)	159 (42.74%)	262 (49.34%)	< 0.001
**Behavioral Care** (%)				
Yes	100 (62.89%)	112 (31.11%)	212 (39.92%)	< 0.001
**Emergency Care** (%)				
Yes	151 (94.97%)	304 (81.72%)	455 (85.69%)	< 0.001

**Table 3:** Adjusted Service Use Estimates Among Individuals with Criminal-Legal Involvement and Documented OUD (n = 147)

	Primary care service use [Odds Ratio (95% CI)]	Behavioral care service use [Odds Ratio (95% CI)]	Emergency department service use [OR (95% CI)]
**Predisposing factors**			
Age	0.99 (0.94–1.04)	1.02 (0.97–1.08)	1.07 (0.96–1.18)
Race (Non-White)	2.09 (0.49–8.95)	1.20 (0.27–5.23)	0.78 (0.06–8.78)
Block group-level indicator unemployment	0.96 (0.89–1.03)	0.96 (0.89–1.03)	0.91 (0.78–1.05)
**Enabling/Disabling factors**			
Health Insurance (Public)	0.25 (0.04–1.61)	0.76 (0.09–5.90)	2.13 (0.21–21.55)
Block group-level indicator of public assistance	1.02 (1.00–1.03)*	1.00 (0.99–1.01)	1.02 (0.99–1.07)
Block group-level indicator of workers commuting by public transit	0.98 (0.95–1.01)	0.99 (0.96–1.02)	1.01 (0.96–1.07)
**Need factors**			
Behavioral Health Disorders			
Depression	1.09 (0.40–3.03)	1.61 (0.55–4.70)	0.56 (0.06–5.05)
Anxiety disorders	8.19 (2.71–24.77)***	23.98 (6.29–91.44)***	5.49 (0.73–40.96)
Non-OUD substance use disorder	4.60 (1.59–13.33)**	6.19 (2.02–18.92)***	0.32 (0.03–3.45)
Chronic Conditions	2.29 (0.56–9.43)	2.10 (0.48–9.20)	0.08 (0.008–0.77)*

Note: Adjusted odds ratios (AORs) and 95% confidence intervals (CIs) estimated using Firth bias-reduced logistic regression. Reference categories: White race; private health insurance; absence of each behavioral health disorder and chronic condition. Age and block group–level indicators (unemployment, public assistance, and workers commuting by public transit) were modeled as continuous variables; odds ratios represent the change in odds associated with a one-unit increase. Chronic conditions represent a combined indicator of HIV, hepatitis C, or any non-communicable chronic disease.

*** p < 0.001

** p < 0.01

* p<0.05.

## Data Availability

The datasets used during the current study are not publicly available due to privacy and HIPAA restrictions but are available from the corresponding author on reasonable request.

## Abbreviations

CLI: Criminal-legal involvement
ED: Emergency department
EHR: Electronic health records
HIPAA: Health Insurance Portability and Accountability Act
HIV: Human Immunodeficiency Virus
MOUD: Medications for Opioid Use Disorder
OUD: Opioid use disorder
SUD: Substance Use Disorder
WHO: World Health Organization
